# Identification of a Novel Splicing Form of Amelogenin Gene in a Reptile, *Ctenosaura similis*

**DOI:** 10.1371/journal.pone.0045871

**Published:** 2012-09-24

**Authors:** Xinping Wang, Xuliang Deng, Xichen Zhang

**Affiliations:** 1 College of Veterinary Medicine, Jilin University, Changchun, Jilin, China; 2 School of Stomatology, Beijing University, Beijing, China; University of Lausanne, Switzerland

## Abstract

Amelogenin, the major enamel matrix protein in tooth development, has been demonstrated to play a significant role in tooth enamel formation. Previous studies have identified the alternative splicing of amelogenin in many mammalian vertebrates as one mechanism for amelogenin heterogeneous expression in teeth. While amelogenin and its splicing forms in mammalian vertebrates have been cloned and sequenced, the amelogenin gene, especially its splicing forms in non-mammalian species, remains largely unknown. To better understand the mechanism underlying amelogenin evolution, we previously cloned and characterized an amelogenin gene sequence from a squamate, the green iguana. In this study, we employed RT-PCR to amplify the amelogenin gene from the black spiny-tailed iguana *Ctenosaura similis* teeth, and discovered a novel splicing form of the amelogenin gene. The transcript of the newly identified iguana amelogenin gene (named *C. Similis*-T2L) is 873 nucleotides long encoding an expected polypeptide of 206 amino acids. The *C. Similis*-T2L contains a unique exon denominated exon X, which is located between exon 5 and exon 6. The *C. Similis*-T2L contains 7 exons including exon 1, 2, 3, 5, X, 6, and 7. Analysis of the secondary and tertiary structures of T2L amelogenin protein demonstrated that exon X has a dramatic effect on the amelogenin structures. This is the first report to provide definitive evidence for the amelogenin alternative splicing in non-mammalian vertebrates, revealing a unique exon X and the splicing form of the amelogenin gene transcript in *Ctenosaura similis*.

## Introduction

As the major enamel matrix protein in tooth development, amelogenin has been demonstrated to play a significant role in tooth enamel formation [1], [2], [3]. Amelogenin has been cloned from many species including human, rat, mouse, hamster, cattle, pig, opossum, snake, platypus, caiman, green *iguana*, *toad,* and *frog*, and its functions have been extensively studied in mammalian vertebrates, especially in mouse, rat, and bovine [4], [5], [6], [7] [1], [8], [9] [10], [11], [12] [13] [14], [15], [16] [17]. It has been reported that mammalian amelogenin exhibits heterogeneity in tooth organs with different isoforms playing different roles in enamel formation [4], [7], [9], [10], [11], [12], [18], [19], [20], [21]. The heterogeneity of amelogenin is reported to be generated either from alternative splicing of amelogenin or posttranslational processing of amelogenin protein [18], [22], [23], [24]. Mammalian amelogenin and its alternative splicing forms have been characterized, splicing forms of amelogenin gene and their functions in non-mammalian species remain largely unknown.

Enamel microstructure between mammalian and non-mammalian vertebrates is significantly different in that mammalian enamel microstructure contains a prismatic, long, and parallel crystallite, while the non-mammalian vertebrate enamel contains prismless, long, and parallel crystallites. One of the mechanisms has been suggested that the complex microstructure observed in many mammals may require a range of amelogenin proteins to yield a variety of HAP crystallites in size and orientation, while the simple enamel microstructure in non-mammalian may require fewer and simpler proteins [25], [26]. To explore the mechanism underlying the enamel microstructure difference among mammalian and non-mammalian vertebrates, and amelogenin evolution, we previously cloned a full-length amelogenin gene and characterized the enamel formation in tooth organs from green iguana. Here, we further report the identification of a novel amelogenin splicing form consisting of 7 exons with a unique exon X in the black spiny-tailed iguana *Ctenosaura similis*.

## Materials and Methods

### Iguana Genomic DNA Isolation

Black spiny-tailed iguana tissue was processed according to the animal protocols proved by Jilin University Laboratory Animal Use and Care Committee. The genomic DNA was isolated using DNeasy blood and tissue kit (Qiagen, Valencia, CA) following the manufacturer’s instruction. The isolated genomic DNA was kept at −80°C for further analysis.

**Figure 1:**
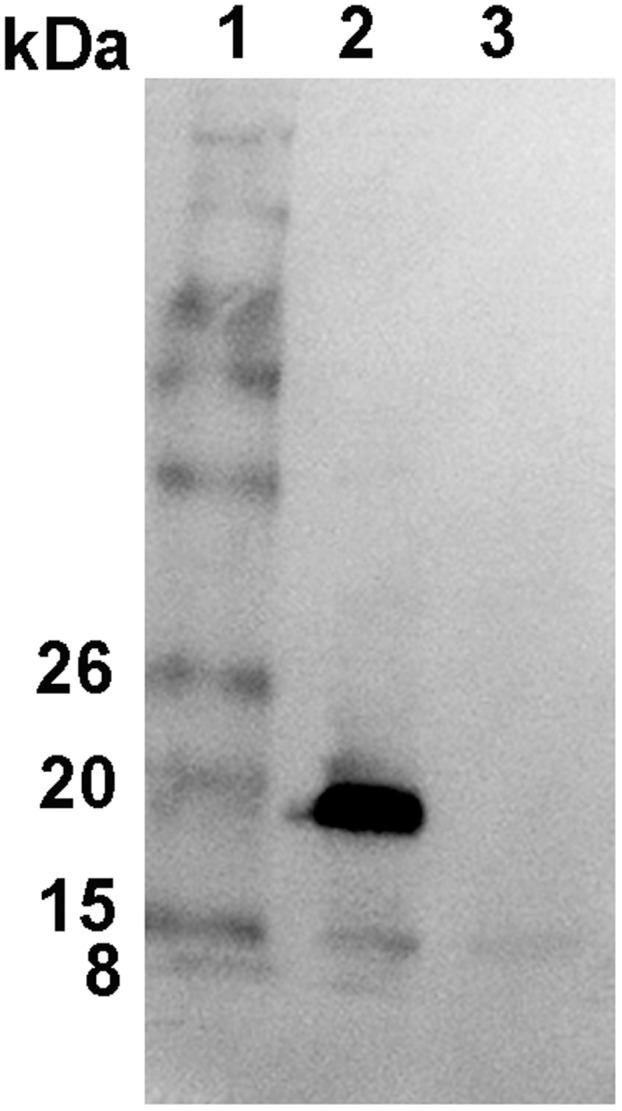
Heterogeneous expression of amelogenin in iguana *Ctenosaura similis* tooth tissue. Detection of amelogenin expression in iguana *Ctenosaura similis* tooth organ using antibody against iguana recombinant amelogenin protein. One major band with molecular weight (MW) of 20 kDa, and two minor bands with MW of 22 kDa and 8 kDa were detected (lane 2), respectively, in relation to proteins marker (lane 1). The non-tooth tissue was used as a negative control (lane 3).

### RNA Isolation and RT-PCR

RNA isolation was performed as previously reported [15]. Briefly, the iguana lizard jaws were removed and homogenized in 1 ml of TRI AGENT® reagent (Sigma Co., St. Louis, Mo), then mixed with 0.2 ml of chloroform and shaken vigorously for 30 sec. The mixture was centrifuged at 12,000×g for 20 min at 4°C. Total RNA was obtained by mixing aqueous phase with equal volume of isopropanol and centrifuging at 12,000×g for 20 min at 4°C. The pellet was washed with 70% ethanol and dissolved in DEPC-treated H_2_O. The isolated RNA was kept at –80°C for further analysis.

**Figure 2:**
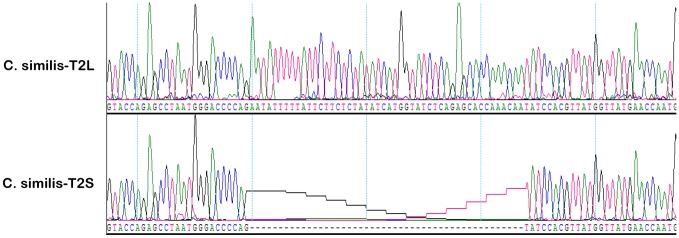
Identification of a novel amelogenin gene splicing transcript in *Ctenosaura similis*. Chromatograms showed the partial sequence of two *Ctenosaura similis* amelogenin transcripts identified by sequencing PCR-amplified products. The cDNA clones corresponding to the transcripts were designed *as C. similis*-T2L (UP) and *C. similis*-T2S (low), respectively. In relation to *C. similis*-T2S, *C. similis*-T2L contains additional nucleotides (UP).

The reverse transcriptase reaction was performed using SuperScript™ II Reverse Transcriptase (Invitrogen, Carlsbad, CA). Briefly, cDNA was synthesized in a volume of 20 µl containing 25 mM Tris-HCI, pH 8.3, 37.5 mM KCI, 1.5 mM MgCI_2_, 5 mM DTT, 0.25 mM each of dATP, dCTP, dGTP and dTTP, 40 units of RNase inhibitor, 200 units of M-MLV reverse transcriptase, 2 µg of total RNA, and 2.5 µM random primers. The cDNA synthesis was carried out at 42°C for 60 min. PCR amplification was done using Taq DNA polymerase (New England Biolabs, Ipswich, MA). The reaction was performed in a total volume of 50 µl containing 20 mM Tris-HCI, pH 8.4, 50 mM KCI, 3 mM MgCI_2_, 0.25 mM each of dATP, dCTP, dGTP and dTTP, 5 unit of Taq DNA polymerase, 1 µM of each primer, and 2 µl of the cDNA synthesized above. The amplification was done under the following conditions: denaturation at 94°C for 5 min; 35 cycles of 94°C for 30 sec, 56°C for 1 min, and 72°C for 1 min; and an additional extension at 72°C for 10 min. The primer sequences were: Rep-UP 5′-ATATCAAGAATCCAAGAAAG-3′; Rep-DN 5′-CTAAATTATTCTTTGCATTA-3′.

**Figure 3:**
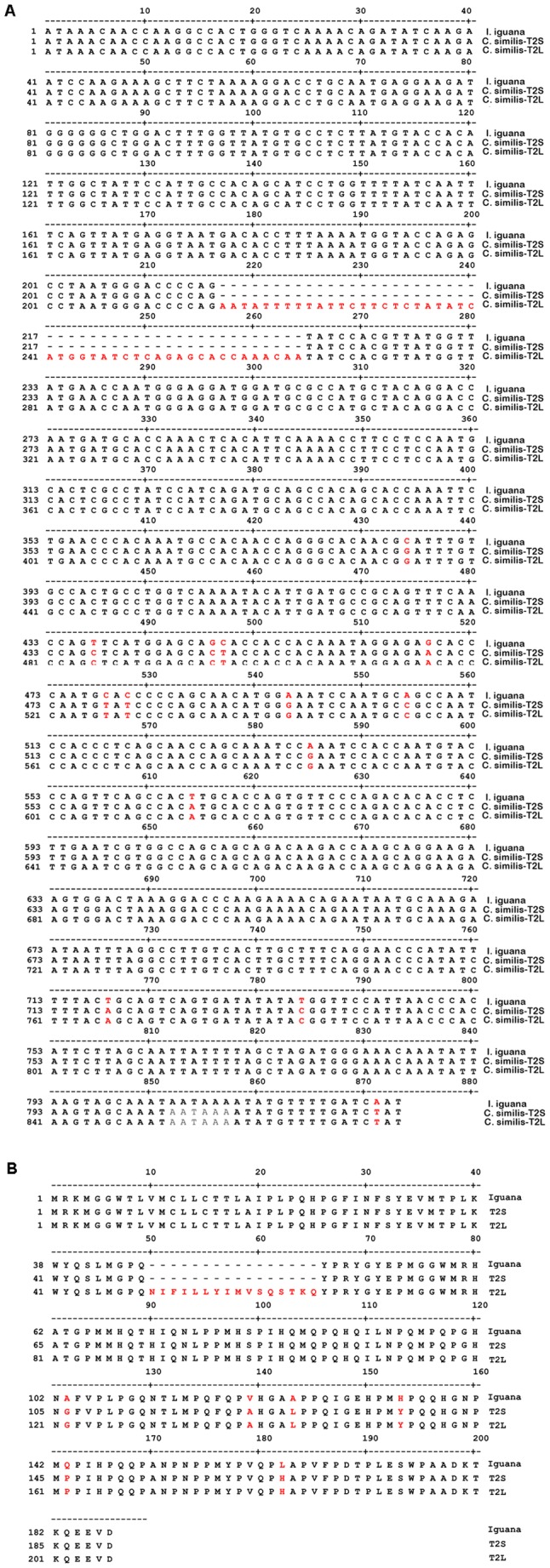
Novel isoforms of amelogenin gene in ***Ctenosaura similis*** **.** (A) Alignment analysis of the full-length novel iguana amelogenin cDNA sequence *C. similis*-T2S, C. *similis*-T2L, and *I. iguana*. The full-length of *C. similis*-T2S transcript is 825 bp, while *C. similis*-T2L transcript is 873 bp. In relation to *C. similis*-T2S and *I. iguana*, the *C. similis*-T2L contains additional 48 bp located between nucleotide 217 and 218. The 5′-untranslated region contains 69 bp upstream of translation start codon ATG. The 3′-untranslated region contains 183 bp. (B) Sequence analysis of deduced amino acid of *I. iguana*, *C. similis*-T2S, and *C. similis*-T2L. The *C. similis*-T2S encodes 190 amino acid residues, while *C. similis*-T2L encodes 206 amino acid residues. Additional 16 amino acid residues were revealed in the deduced *C. similis*-T2L amino acid sequence located between amino acid residue 49 and 50 in relation to *C. similis*-T2S. Similar to *I. iguana*, the T2S and T2L also have a deletion of 3 amino acid residues in exon 3. The nucleotides/amino acid sequence variations were indicated and highlighted as red color.

**Figure 4:**
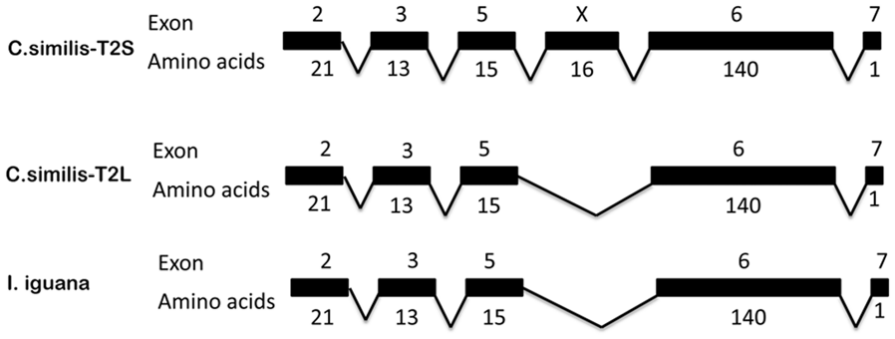
Novel iguana amelogenin gene cDNA structure. Analysis of novel iguana amelogenin sequence revealed the full-length amelogenin cDNA containing 7 exons including exon 1, 2, 3, 5, X, 6, and 7 (exon numbers is relative to published mammalian amelogenin exon numbers). Different from amelogenin genes of all species so far, an unique exon named exon X between exon 5 and exon 6 was detected; Similar to other species, no corresponding sequence elements were detected resembling exon 4, suggesting that amelogenin exon 4 is skipped or deleted in *Ctenosaura similis*.

### Cloning and Sequencing

PCR products were analyzed by electrophoresis using 1% agarose gel and cloned to pGEM-T vector (Promega, Madison, WI). Recombinants were confirmed by sequencing (Sangon Biotech, Shanghai). The resulting sequences were analyzed using DNAstar Lasergene software. The nucleotide sequence served as a template for searching homologous sequences through GenBank (www.ncbi.nlm.nih.gov). Alignment analysis of multiple sequences was performed using the Clustal W method [27]. The amelogenin amino acid sequence was deduced from the nucleotide sequence. The hydrophilicity-plots were generated using the Kyte and Doolittle algorithm [29]. The protein secondary structures were predicted using Psipred [30]. The protein homology detections and structure predictions were performed by HMM-HMM comparison using HHpred [31].

**Table 1:** Primers used for determination of the exon and intron boundaries.

Primers	Sequence (5′-3′)	Orientation
Ex1S	CCAAGGCCACTGGGTCAAAACAGATATC	S
Ex2A	GTGGTACATAAGAGGCACATAACCAAAGTCC	A
Ex2S	GGACTTTGGTTATGTGCCTCTTATGTACCAC	S
Ex3A	CTGAAATTGATAAAACCAGGATGCTGTGG	A
Ex3S	CCACAGCATCCTGGTTTTATCAATTTCAG	S
Ex5A	GGGTCCCATTAGGCTCTGGTACCATTTTAAAGG	A
Ex5S	CCTTTAAAATGGTACCAGAGCCTAATGGGACCC	S
ExXA	GCTCTGAGATACCATGATATAGAG	A
ExXS	CTCTATATCATGGTATCTCAGAGC	S
Ex6A	CATTGGTCCTGTAGCATGGCGCATCCATCC	A
Ex6S	TCGTGGCCAGCAGCAGACAAGACCAAGCAGG	S
Ex7A	GCAAGTGACAAGGCCTAAATTATTCTTTGC	A

S refers to sense; A stands for antisense.

**Table 2:** Squamate *Ctenosaura similis* amelogenin gene exon-intron sequence organization.

Size			Sequence of exon-intron junction
Exon	Exon(bp)	Intron (kb)	Splice donor Splice acceptor
1	61	2.0	AAAG gtaaatgtatactt…….ttacag GAC
2	71	1.65	TCCA gtaagtactagtta…….ctacag TTG
3	39	0.69	TGAG gtaccattgttttt…….cccaag GTA
5	45	1.7	CCAG gtatgtcctctata…….tattag AAT
X	48	0.46	ACAA gtaagaccaattta…….taaaag TAT
6	420	1.4	AGAA gtaagaatttctgt…….ttccag GAC
7	241		

Exon and intron sequence are indicated in the upper-case and low-case letter, respectively.

### 5′- and 3′-RACE

5′- and 3′-RACE was performed to determine the 5′- and 3′-ends of the amelogenin gene sequence following a standard protocols. The full-length of amelogenin gene sequence was reconstructed with 5′-and 3′-end sequences, further amplified with primers Rep-UP/Rep-DN using Failsafe premix PCR system (Epicentre, Madison, WI) following manufacturer’s instruction, and confirmed by DNA sequencing.

**Figure 5:**
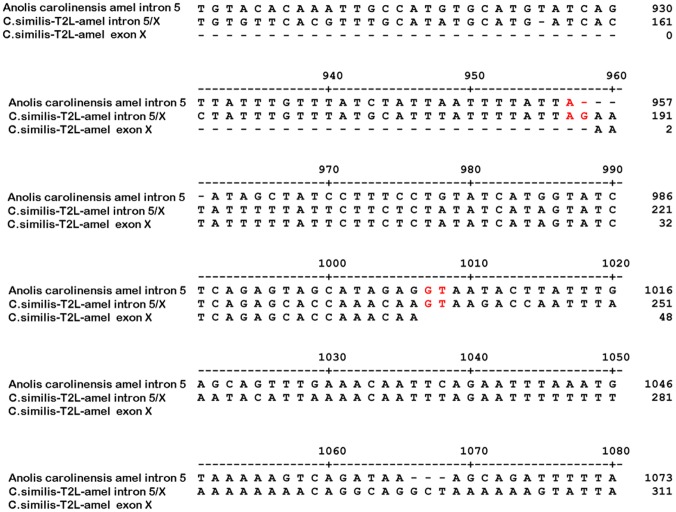
Alignment of genomic sequences spanning exon X of *C. similis*-T2L amelogenin gene with the selected species. The black spiny-tailed iguana genomic sequence spanning the exon X was amplified by PCR and analyzed by alignment with the Carolina anole in the same region of the black spiny-tailed iguana exon X sequence. Like the majority of exons, a conserved sequence feature, the presence of AG at the 5′ splice site and GT at the 3′ splice site were observed spanning the exon X, while the same sequence region of the Carolina anole share a high sequence identity except the lack of G in AG at the 5′ splice site, which fail to generate a exon X observed in the black spiny-tailed iguana. The 5′ and 3′ splice sites in the black spiny-tailed iguana genomic sequence spanning the exon X are highlighted as red. Alignment gaps are indicated by a dash (−).

### Western-blot

Western-blot assay for detection of amelogenin expression in the black spiny-tailed iguana teeth was performed following the protocol described previously [16]. Briefly, iguana teeth were homogenized, harvested, washed twice with phosphate-buffered saline, pelleted, and suspended in 400 µl of sodium dodecyl sulfate sample buffer. After protein quantitation, 100 µg of protein for each sample was then subjected to12% SDS-PAGE gel before proteins were transferred to nitrocellulose membrane (Schleicher & Schuell, Keene, NH). The immunoblot was blocked in TBST with 5% non-fat milk, reacted with primary antibodies against iguana recombinant amelogenin, then followed by probing with a 1∶5000 dilution of secondary antibody against rabbit immunoglobulin conjugated with alkaline phosphatase (Sigma) and detected by color-development in NBT/BCIP substrate (Stratagene). The black spiny-tailed iguana tail tissue was processed and used as amelogenin-negative sample control.

**Figure 6:**
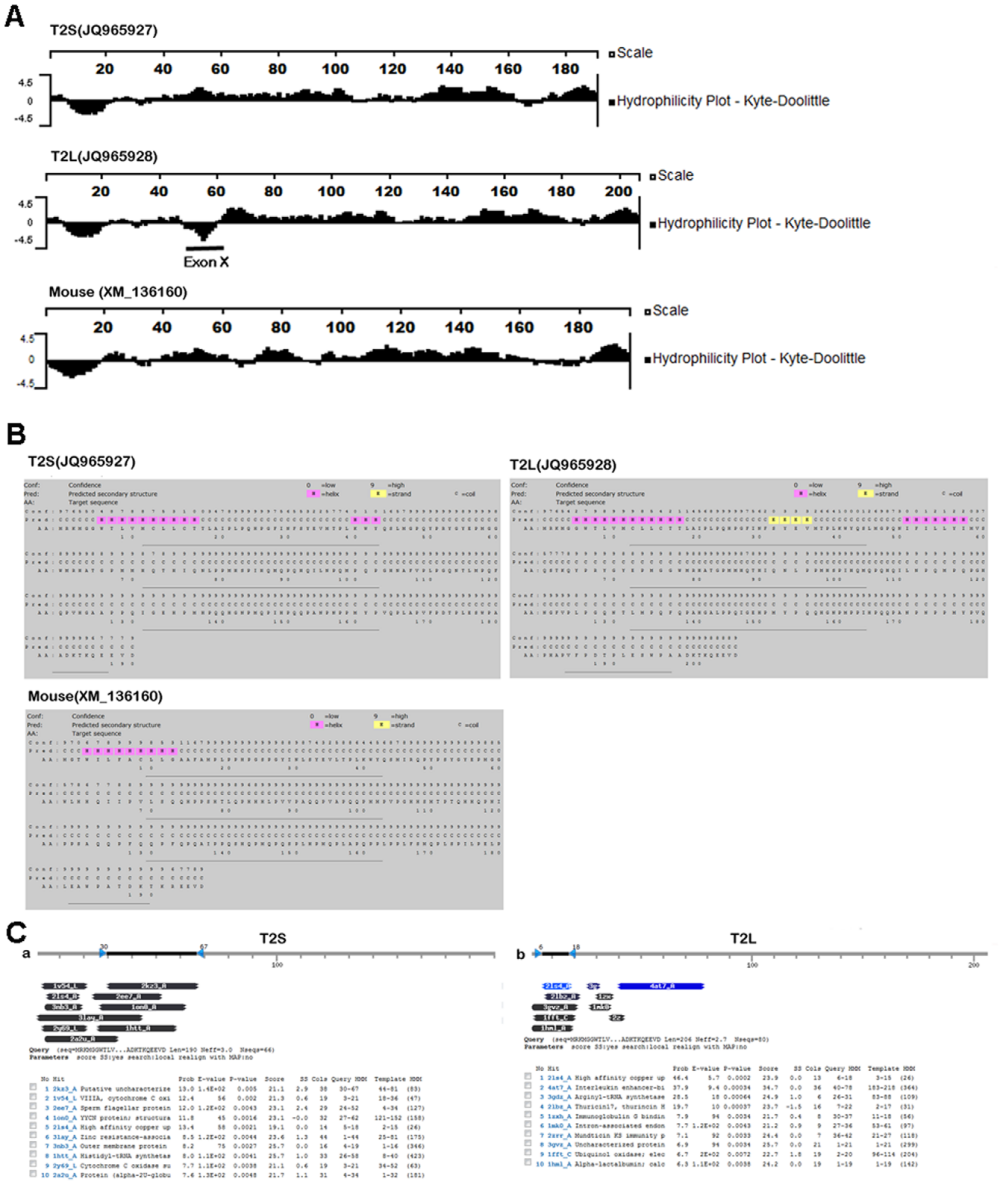
Effect of exon X sequence on the amelogenin protein structures. (A) The hydrophilicity-plot analysis using the Kyte and Doolittle algorithm. The hydrophilicity-plots of T2S and T2L and mouse (XM_136160) were generated and compared. In relation to T2S and mouse amelogenin, the region underlined with black line (middle panel) is exon X sequence, which is highly hydrophobic. (B) Exon X affects the secondary structure of T2L predicted by Psipred. Two potential helixes regions for T2S (aa 7∼16; aa 41∼43) and T2L (aa 6∼17; aa 51∼57) in relation to one potential helix region for mouse amelogenin (aa 4∼12) were revealed by Psipred prediction. A strand region (aa 32–35) was also revealed in relation to T2S and mouse amelogenin. (C) Exon X sequence effects on the tertiary structures of T2L. T2S and T2L were used as query sequence for homology detection and structure prediction by HMM-HMM comparison using HHpred. A bar graph summarizes the positions and color-coded significances of the database matches with the probability. A tabular hit lists with probabilities, E-values, scores, and match regions in query and templates.

### H&E Staining and Immunohistochemistry

Tooth tissue was fixed in 10% buffered formalin and processed for paraffin histology. Sections were either stained with hematoxylin and eosin to reveal the tooth organ structure or probed with primary antibody against recombinant iguana amelogenin or antibody against newly defined exon X peptide (NIFILLYIMVSQSTKQ) for determination of amelogenin protein expression. The immunohistochemistry assay was performed as previously described [16].

**Figure 7:**
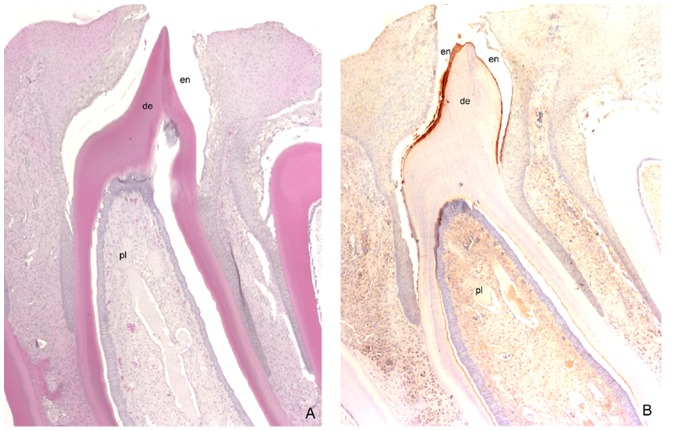
Expression of exon X protein in tooth organ. ( A) The microphotograph illustrates the components of the developing iguana tooth organ, including the enamel layer (en), dental layer (de) and dental pulp (pl). (B) Expression of amelogenin protein isoform containing exon X in the *Ctenosaura similis* tooth organs by immunohistochemistry staining using antibody against iguana amelogenin exon X peptide. Immunoreaction with antibody against exon X was detected in the enamel layer (en) and dental pulp.

## Results

### Heterogeneous Amelogenin Expression in Iguana Tooth Organs

Alternative splicing of amelogenin gene in mammalian tooth organs has been extensively studied and demonstrated to be a major mechanism of amelogenin heterogeneity [11], [20], [28]. Previously, we identified and characterized a full-length cDNA sequence of the amelogenin gene from a green iguana, and observed amelogenin protein expression in the green iguana tooth organs by western blot assays and immunohistochemistry [15]. To expand this observation, we employed a Western blot to detect the amelogenin expression in the black spiny-tailed iguana tooth organs using newly generated antibody against recombinant amelogenin protein. As shown in Figure 1, one band with a molecular weight about 15 kDa was revealed in both tooth organs and non-tooth tissue from the black spiny-tailed iguana, suggesting this band is a non-specific one. Compared with non-tooth tissue control, one dominant band with a molecular weight about 20 kDa, and two minor faint bands with molecular weight about 21 kDa and 8 kDa were detected, respectively, indicating a heterogeneous amelogenin expression in *Ctenosaura similis* tooth organs.

### Identification of Novel Amelogenin Gene Splicing form in *Ctenosaura similis*

To explore the mechanism underlying the heterogeneous amelogenin protein expression in the black spiny-tailed iguana tooth organs, we designed the primers based on the available published amelogenin sequence in an attempt to amplify the potential splicing forms in *Ctenosaura similis* teeth organs using RT-PCR. After amplification, the PCR products were cloned into pGEM-T vector and recombinants were sent out for sequencing. Sequence analysis revealed two different amelogenin clones, one designed as *C. similis-*T2S, another designed as *C. similis*-T2L. In relation to *C. similis*-T2S, the *C. similis*-T2L contained additional nucleotides, suggesting the expression of different transcripts in the iguana tooth organs (Figure 2).

To characterize the newly identified two transcripts of the black spiny-tailed iguana amelogenin, we used the 5′- and 3′- RACE to obtain the end sequence of amelogenin mRNAs. Full-length amelogenin cDNA sequence was reconstructed by joining the sequences of a single 5′ end and 3′ end, and further confirmed with primers Rep-UP/Rep-DN for amplifying the complete amelogenin cDNA sequence using Failsafe premix PCR. Sequence results demonstrated that the full-length *C. similis*-T2S transcript contained 825 nucleotides, encoding 190 amino acid residues (GenBank accession number JQ965927), while full-length *C. similis*-2TL transcript consisted of 873 nucleotides, encoding 206 amino acid residues (GenBank accession number JQ965928) (Figure 3). The 5′-untranslated region was 69 bp upstream of the translation start codon ATG, the 3′-untranslated region was 183 bp downstream from the stop codon. The amelogenin proteins encoded by *C. similis*-T2S and *C. similis*-T2L transcripts were designed as T2S and T2L, respectively.

### Unique Exon X in *C. similis*-T2L Transcript of Amelogenin Gene

To define the additional sequence observed in *C. similis*-T2L, alignment analysis of *C. similis*-T2L nucleotide sequence with *C. similis*-T2S and a previously obtained green iguana amelogenin sequence (designed as *I. iguana*) was performed. In relation to *C. similis*-T2S and *I. iguana*, *C. similis*-T2L contained an additional 48 nucleotides (Figure 3A). The 48 nucleotides are located between nucleotide 216 and nucleotide 217 of *C. similis*-T2S transcript, encoding 16 amino acid residues. Analysis of the amelogenin gene organization of *C. similis*-T2L revealed that the 16 amino acid residues match no amelogenin exon sequence in comparison with the amelogenin sequence from other species available so far. In relation to the current amelogenin gene structure, the 16 amino acid residues are located between exon 5 and exon 6, it is a novel exon and named exon X. As shown in Figure 4, the *C. similis*-T2L amelogenin gene consists of 7 exons including exon1, 2, 3, 5, X, 6, and 7.

To further confirm that exon X is truly a novel amelogenin exon, the genomic DNA sequence from the black spiny-tailed iguana was isolated and used as template to amplify the sequence spanning the exon X using the primer pair for amplification of the amelogenin exon 5/exon 6 boundaries (table 1). As shown in table 2, sequence analysis of the 2.2 kb PCR product identified the boundary sequences of exon 5/exon 6 and exon 6/exon 7. Further analysis of this fragment revealed the boundaries between exon 5/exon X and exon X/exon 6. The intron region between exon 5/exon X is about 1.7 kb, while the intron sequence between exon X/exon 6 is 454 bp, demonstrating the exon X is indeed a novel amelogenin exon.

Since the exon X was confirmed to be the unique exon in amelogenin gene, we utilized sequence comparison to explore the origin of exon X and determine whether exon X has any homolog in other species. Analysis of the genomic sequence spanning the exon X in the black spiny-tailed iguana with the corresponding areas of several amelogenin gene sequences including human (CR627366), bovine (AC_000187), rat (NC_005120), mice (NC_000086), and the Carolina anole (NW_003339249) was performed. The sequences of intron 5 of amelogenin gene in mammalian vertebrates were revealed to be short, from 180 bp to 280 bp, while the sequences of intron 5 in non-mammalian vertebrates were much longer, normally from 1.2 kb to 2.2 kb (not shown). Analysis of the above mammalian amelogenin genes showed no splice donor/acceptor sequence feature within intron 5 sequence (not shown). As shown in Figure 5, like the majority of exons, the exon X retained the conserved sequence feature of the presence of AG at the 5′ splice site and GT at the 3′ splice site (table 2), while the Carolina anole, a lizard with high identity of amino acid sequence with the black spiny-tailed iguana and high nucleotide sequence identity spanning the exon X region, showed a lack of nucleotide G in AG of the 5′ splice site, which is likely responsible for its skipping exon X observed in *C. similis*-T2L amelogenin gene.

### Exon X Sequence Affects T2L Protein Structures

To determine whether exon X has any effect on the amelogenin protein structure, the hydrophilicity-plots of T2S and T2Lwere generated using the Kyte and Doolittle algorithm, and secondary structures of T2S and T2L were predicted using Psipred. As shown in Figure 6A, in relation to the hydrophilicity plot of T2S and mouse amelogenin (XM_136160), the exon X sequence (AA 50∼65) of T2L turned out to be hydrophobic, which dramatically affected its N-terminal hydrophilicity-plot. Psipred prediction revealed a two potential helix regions for T2S (AA 7∼16; AA 41∼43) and T2L (AA 6∼17; AA 51∼57) in relation to one potential helix region for mouse amelogenin (AA 4∼12) (Figure 6B). In addition, T2L also showed a strand region (AA 32–35). Those results suggest that exon X has a dramatic effect on its secondary structure.

To further determine the potential effect of exon X sequence on the tertiary structures of T2L, T2S and T2L were used as a query sequences for protein homology detection and structure prediction by HMM-HMM comparison using HHpred. As shown in Figure 6C, representing proteins with closest homologs of structure or domains with T2S (a) or T2L (b) were listed. The majority of listed homologs with T2L are different from those of T2S, indicating that exon X sequence also affects the tertiary structure of T2L.

Taken together, the above results suggest that exon X is likely to affect the T2L structure, thus the functions of this novel splicing form of amelogenin.

### Expression of T2L in Iguana *Ctenosaura similis* Tooth Organs

To determine the expression pattern of T2L amelogenin protein, the antibody against the exon X peptide (NIFILLYIMVSQSTKQ) was generated and applied to localize the expression of amelogenin protein containing exon X in the tooth organs. As documented in Figure 7A, the black spiny-tailed iguana tooth organs clearly showed the components of the developing tooth including the enamel layer, dentin layer and dental pulp. Similar to the immunoreaction of the polyclonal antibody against recombinant iguana amelogenin antigen (not shown), a strong signal were detected in the black spiny-tailed iguana tooth enamel layer when the antibody against exon X was applied (Figure 7B), suggesting that both T2L and T2S were expressed in *Ctenosaura similis* tooth organs.

## Discussion

In this study, we have demonstrated the heterogeneous amelogenin expression in the black spiny-tailed iguana tooth organs using western blot and immunohistochemistry, discovered for the first time the alternative splicing of amelogenin gene in non-mammalian vertebrate by employing RT-PCR and sequencing analysis. The results in this study revealed one predominant band with the molecular weight about 20 kDa, and two minor bands with molecular weight about 21 kDa and 8 kDa, respectively, indicating the heterogeneous amelogenin expression in iguana tooth organs. The detection of amelogenin expression using antibodies against recombinant iguana amelogenin protein and exon X peptide in tooth organs further confirms our finding of amelogenin heterogeneous expression in the black spiny-tailed iguana. As a major tooth enamel matrix protein, amelogenin protein has been demonstrated to play a crucial role in the enamel formation and functions as a signaling molecule during tooth biomineralization. The discovery of two different splicing forms of amelogenin gene in the black spiny-tailed iguana suggest that amelogenin alternative splicing, as one of the mechanisms of amelogenin heterogeneity in tooth organs, is repeatedly employed from non-mammalian to mammalian vertebrates.

Heterogeneous amelogenin protein expression in mammalian tooth organ is believed to be the result from either alternative splicing or posttranslational proteolysis of amelogenin. In this study, we employed PCR and sequence analysis to discover two amelogenin gene splicing forms in the black spiny-tailed iguana teeth. Sequencing provides definitive evidence showing that *C. similis*-T2S and *C. similis*-T2L are different splicing forms of transcripts expressed in iguana tooth organ. These findings are congruent with amelogenin protein expression pattern detected in tooth organs. Amelogenin, the major enamel matrix protein of the developing tooth organ, is highly conserved throughout most species studied. Amelogenin gene structure is similar, normally consisting of 7 exons including exon 1, 2, 3, 4, 5, 6, and 7 in which exon 4 was usually skipped during the processing of amelogenin pre-mRNA. Exon 6 is the largest exon of amelogenin gene in which four domains named exon 6A, exon 6B, exon 6C, and exon 6D were further divided. The sequence of exon 6A, C, and D is relatively conserved, while exon 6B is relatively variable. In comparison to *I. iguana*, the *C. similis*-T2S and *C. similis*-T2L in this study share a very high amino acid sequence identity in the region from exon 2 to exon 6A, and the N-terminal of exon 6B, while there are some variations of amino acid sequence in the C-terminal of exon 6B. Those results indicate that the black spiny-tailed iguana amelogenin gene exon 6B, similar to observations in most species studied, is a relatively variable region. Like the observation in *I. iguana* in which the exon 3 lacks of 3 amino acid residues that significantly affect the hydrophilicity of the N-terminal portion of matured amelogenin protein in *Iguana* iguana, the black spiny-tailed *C. similis*-T2S/T2L amelogenin transcripts also contains an exon 3 encoding just 13 amino acid residues, which is different with the majority of exon 3 sequence reported in other species. Like the majority of amelogenin gene in other species, no exon 4 was detected in the black spiny-tailed iguana.

The exon X revealed in this study consists of 48 nucleotides encoding 16 amino acid residues. Sequence analysis showed no match of exon X to any amelogenin exon sequence, suggesting it is a novel exon. This novel exon is located between nucleotide 216 and nucleotide 217 in comparison to *C. similis*-T2S nucleotide sequence. In relation to the amelogenin gene structure, exon X is located just between exon 5 and exon 6.

Several lines of evidence in this study clearly exclude the possibility that exon X is an artifacts generated during cDNA synthesis. First, sequencing results clearly showed the amplification of the *C. similis*-T2S and *C. similis*-T2L cDNA clones in the same iguana tooth organs. Second, sequencing the genomic DNA fragment with primer covering intron 5 (exon5/exon 6) identified the exon X sequence and revealed a typical splicing donor/acceptor sites spanning exon X. Third, analysis of this fragment defined the boundaries between exon 5/exon X and exon X/exon 6 (table 2). The intron region between exon 5/exon X is about 1.7 kb, while the intron sequence between exon X/exon 6 is 454 bp, demonstrating that the exon X is indeed a novel exon in amelogenin gene. According to the current amelogenin gene structure, *C. similis*-T2L consists of 7 exons (exon 1, 2, 3, 5, X, 6, and7). The discovery of novel exon X in iguana amelogenin gene provides important evidence underlying the amelogenin evolution, and will open up a new avenue for the exploration of amelogenin evolution.

The demonstration that exon X is the novel amelogenin exon instantly attracted us to explore whether exon X has any homolog in other species. Analysis of the amelogenin gene corresponding genomic sequence (intron 5) of several mammalian and non-mammalian vertebrates revealed that lengths of intron 5 of amelogenin gene in mammalian vertebrates were from 180 bp to 280 bp, while the sequences of intron 5 in non-mammalian vertebrates were normally from 1.2 kb to 2.2 kb. The intron 5 sequences in mammalian vertebrates are significantly smaller than those of reptilian and amphibian. Analysis of the mammalian amelogenin genes including human, rat, mice, and bovine showed no splice donor/acceptor sequence feature within their intron 5 sequence. However, sequence comparison analysis of the amelogenin gene intron 5 of the black spiny-tailed iguana with another known lizard, the Carolina anole clearly showed the high sequence identity within intron 5, especially the sequence spanning the exon X. It is interesting to note the lack of the acceptor splice site and the presence of the donor splice site in the corresponding genomic sequence of the Carolina anole. The results that exon X, like the majority of exons, retains the conserved sequence feature by the presence of AG at the 5′ splice site and GT at the 3′ splice site, while the Carolina anole with just a lack of nucleotide G in AG at the 5′ splice site, significantly changed the outcome of amelogenin gene splicing, indicating the sequence surrounding the splice site will dramatically affect the gene splicing.

A recent study has identified 4 major amelogenin protein structural motifs/domains from the full-length mouse amelogenin protein using NMR spectroscopy [32]. Those 4 functional domains include an N-terminal TRAP domain (AA 1∼45), a central histidine-rich region (AA 46∼125), the proproline tripeptide repeat region (AA 126∼164), and the hydrophilic charged C–terminal (AA 165∼180). We analyzed the hydrophilicity-plots of T2L using the Kyte and Doolittle algorithm and find exon X to be hydrophobic in relation to T2S and mouse amelogenin, indicating that exon X might play a role in the T2L structure change. The secondary and tertiary structures predicted using Psipred and HHpred for T2L were different from those of T2S and mouse amelogenin, indicating that exon X is likely to affect the structure of T2L, even its function(s). Although different programs for predicting the secondary and tertiary structure of T2S, T2L and mouse (XM_136160) were performed, our results failed to yield the same pattern, even in the full-length mouse amelogenin as reported by Zhang’s group. This might be due to the different programs we used. The exon X detected in black spiny-tailed iguana from this study will contribute to the understanding of tooth enamel evolution by revealing the conservation and divergence of significant exons of the amelogenin gene throughout vertebrate evolution. Unveiling the underlying mechanism relating to the iguana amelogenin splicing, the origin of exon X, and the functional study of exon X are subjects of future investigation.
